# The influence of HLA‐DRB1*15 on the relationship between microglia and neurons in multiple sclerosis normal appearing cortical grey matter

**DOI:** 10.1111/bpa.13041

**Published:** 2021-12-13

**Authors:** Richard L. Yates, Jonathan Pansieri, Qizhu Li, Jack S. Bell, Sydney A. Yee, Jacqueline Palace, Margaret M. Esiri, Gabriele C. DeLuca

**Affiliations:** ^1^ Nuffield Department of Clinical Neurosciences University of Oxford Oxford UK; ^2^ Department of Engineering Science University of Oxford Oxford UK; ^3^ Salford Royal NHS Foundation Trust Salford UK

**Keywords:** cortex, microglia, multiple sclerosis, neurodegeneration, neuron, pathology, synapse

## Abstract

Cortical tissue injury is common in multiple sclerosis (MS) and associates with disability progression. We have previously shown that HLA‐DRB1*15 genotype status associates with the extent of cortical inflammatory pathology. In the current study, we sought to examine the influence of HLA‐DRB1*15 on relationships between inflammation and neurodegeneration in MS. Human post‐mortem MS cases (*n* = 47) and controls (*n* = 10) were used. Adjacent sections of motor cortex were stained for microglia (Iba1+, CD68+, TMEM119+), lymphocytes (CD3+, CD8+), GFAP+ astrocytes, and neurons (NeuN+). A subset of MS cases (*n* = 20) and controls (*n* = 7) were double‐labeled for neurofilament and glutamic acid decarboxylase 65/67 (GAD+) to assess the extent of the inhibitory synaptic loss. In MS cases, microglial protein expression positively correlated with neuron density (Iba1+: *r* = 0.548, *p* < 0.001, CD68+: *r* = 0.498, *p* = 0.001, TMEM119+ *r* = 0.437, *p* = 0.003). This finding was restricted to MS cases *not* carrying HLA‐DRB1*15. Evidence of a 14% reduction in inhibitory synapses in MS was detected (MS: 0.299 ± 0.006 synapses/μm^2^ neuronal membrane versus control: 0.348 ± 0.009 synapses/μm^2^ neuronal membrane, *p* = 0.005). Neurons expressing inhibitory synapses were 24% smaller in MS cases compared to the control (MS: 403 ± 15 μm^2^ versus control: 531 ± 29 μm^2^, *p* = 0.001), a finding driven by HLA‐DRB1*15+ cases (*15*+: 376 ± 21 μm^2^ vs. *15*−: 432 ± 22 μm^2^, *p* = 0.018). Taken together, our results demonstrate that HLA‐DRB1*15 modulates the relationship between microglial inflammation, inhibitory synapses, and neuronal density in the MS cortex.

## Graphical abstract

Cortical tissue injury is common in multiple sclerosis and associates with disability progression. We have previously shown that HLA‐DRB1*15 genotype status associates with the extent of cortical inflammatory pathology. In the current study, we extend these findings to show that carriage of HLA‐DRB1*15 influences relationships between microglia and neurons in the cortical normal appearing grey matter.

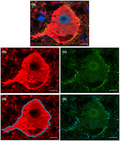

## 1 INTRODUCTION

Cortical tissue injury contributes to the progression of irreversible disability in multiple sclerosis (MS) [1, 2]. Neuronal loss is a feature of MS cortical pathology, but reports are conflicting as to its distribution, extent, and determinant pathological pathways [3, 4, 5]. It has been demonstrated that significant neuronal loss occurs in the superficial cortical layers of MS cases harboring tertiary lymphoid‐like follicles in the overlying meninges [6]. This work has led to a cascade of studies that have cast blame on diffusible cytotoxic factors emanating from the subarachnoid space [7, 8, 9], as well as an intrinsic vulnerability of neurons in superficial cortical layers [10]. However, it is notable that despite substantial meningeal inflammation that is present from the earliest phases of the disease [8], and continuing over decades [7], that the vast majority of cortical neurons survive the MS disease process [4]. In particular, neuronal loss in deeper cortical layers is minimal in MS compared to non‐neurological controls despite the presence of a chronic parenchymal inflammatory milieu [6]. However, differences in neuronal loss emerge when segregating MS cases by the extent of fibrinogen deposition, with cases demonstrating a higher burden showing greater neuronal loss [11]. As fibrinogen is a well‐described and potent activator of microglia, the relationship between microglia and neuronal loss warrants evaluation.

The relationship between microglial inflammation and neurodegeneration in the MS cortex is likely to be complex. In vitro studies have demonstrated a marked phenotypic heterogeneity in microglia, with neuroprotective responses described [12]. Animal models have demonstrated that activated microglia strip inhibitory synapses from neurons exposed to inflammatory insult to confer neuroprotection [13]. Removal of inhibitory synapses blunts neuronal apoptosis via upregulated expression of pro‐survival genes induced by enhanced excitatory neurotransmission [13]. To add to this complexity, recent work from our group has shown that carriage of the primary genetic risk factor for MS, HLA‐DRB1*15, influences the extent of cortical microglial inflammation in younger cases [14, 15]. However, the association of HLA‐DRB1*15 with microglial phenotypes relevant to neuronal survival in functionally relevant deep cortical layers in MS remains unknown. To this end, we sought to examine relationships between parenchymal inflammation, inhibitory synapses, and neurons in the MS cortex, and the role of HLA‐DRB1*15 therein.

In the current study, we provide evidence that microglial protein expression positively associates with neuronal density in MS normal appearing grey matter (NAGM), a relationship not seen in cases carrying the HLA‐DRB1*15 allele. We also report a selective reduction of inhibitory synapses in the MS cortex and an influence of HLA‐DRB1*15 on the extent of neuronal atrophy in MS cases. Our work casts light on heterogeneity between MS cases that might not be appreciated with post‐mortem evaluation of MS cases without the consideration of the HLA‐DRB1*15 allele. The fact that microglia relate differently to neuronal density depending on HLA‐DRB1*15 genotype status sets the stage for identifying therapeutic targets that exploit the beneficial potential of these fascinating cells.

## 2 MATERIALS AND METHODS

### 2.1 Study population

Human archival material of MS (*n* = 47) and control cases (*n* = 10) from the UK MS tissue bank, Imperial College, London (Research Ethics Code [REC] 08/MRE09/3115), and Oxford Brain Bank, Oxford (REC 07/0606/85) was used with ethical approval as per Human Tissue Act 2006 guidelines (Table 1). The primary motor cortex was sampled from the mesial precentral gyrus and MS cases were previously genotyped for HLA‐DRB1*15 status, as described [14]. The MS cases used have been the subject of a previous study [14], albeit compared to a different control cohort caused by tissue availability for downstream analyses.

**TABLE 1:** Clinical details of MS and control cohort

	MS cases (*n* = 47)	*HLA*‐DRB1*15+ (*n* = 21)	*HLA*‐DRB1*15− (*n* = 26)	Controls (*n* = 10)
Age of death (yr)	63 (range: 40–92)	65 (range: 40–92)	62 (range: 43–86)	74 (range: 57–91)
Duration of disease (yr)	31 (range: 11–58)	32 (range: 12–58)	30 (range: 11–55)	n/a
Sex	M = 13; F = 34	M = 7; F = 14	M = 6; F = 20	M = 7; F = 3
Clinical course	PPMS: 4; SPMS: 38; RRMS: 1; unknown: 4	PPMS: 3; SPMS: 17; RRMS: 0; unknown: 1	PPMS: 1; SPMS: 21; RRMS: 1; unknown: 3	n/a
Brain weight (g)	1157 (range: 894–1380)	1163 (range: 1000–1364)	1152 (range: 894–1380)	1320 (range: 1072–1628)
PM interval (h)	18 (range: 7–38)	18 (range: 7–38)	18 (range: 8–28)	36.7 (range: 10–72)

Note

Values represent the mean unless stated otherwise.

Abbreviations: n/a, not applicable; PM, post‐mortem; PPMS, primary progressive MS; RRMS, relapsing‐remitting MS; SPMS, secondary progressive MS.

### 2.2 Immunohistochemistry and immunofluorescence

Formalin‐fixed paraffin‐embedded tissue blocks were cut into 6‐µm thick sections and labeled with primary antibodies for myelin (PLP), microglia/macrophages (Iba1), activated microglia/macrophages (CD68), resident microglia (TMEM119), T‐lymphocytes (CD3), cytotoxic T‐lymphocytes (CD8), GFAP+ astrocytes, and neurons (NeuN) using DAB immunohistochemistry, as previously described (Table 2) [11]. Sections were counterstained with hematoxylin for 1 min. Experiments with the omission of primary antibodies were used as negative controls.

**TABLE 2:** Details of antibodies used and immunohistochemical and immunofluorescence methods

Target	Primary antibody	Antibody dilution	Clone	Antigen retrieval	Incubation settings
PLP	AbD serotec #MCA839G	1: 1000	Monoclonal	Citrate pH6 microwave	1h RT
Iba1	Wako #019‐19741	1: 1000	Polyclonal	Citrate pH6 Autoclave	1h RT
CD68	Dako #M087601‐2	1: 100	Monoclonal	Citrate pH6 Autoclave	1h RT
TMEM119	Sigma #HPA051870	1: 1500	Polyclonal	Tris‐EDTA pH 9 Autoclave	ON 4°C
CD3	Dako #A0452	1: 100	Polyclonal	Tris‐EDTA pH 9 Autoclave	1h RT
CD8	DAko #IS623	1:5	Monoclonal	Citrate pH6 Autoclave	1h RT
GFAP	Dako Cytomation #Z0334	1: 8000	Polyclonal	Citrate pH6 microwave	1h RT
NeuN	Milipore #MAB377	1: 400	Monoclonal	Citrate pH6 Autoclave	1h RT
Neurofilament	Sigma #N0142	1/500	Monoclonal	Citrate pH6 microwave	ON 4°C
GAD 65/67	Abcam #ab1511	1/1000	Polyclonal	Citrate pH6 Autoclave	ON 4°C
Synaptophysin	Abin #350897	1/300	Polyclonal	Citrate pH6 Autoclave	ON 4°C

Abbreviations: ON, overnight; RT, room temperature.

Synaptic coverage of neurons was assessed using a primary antibody for GAD 65/67 for inhibitory synapses (hereafter referred to simply as “GAD”), and to synaptophysin for total synapses. Each was assessed independently by labeling sections with primary antibodies to either GAD or synaptophysin, and then double‐labeling for neurons (neurofilament), conjugated, respectively, to Alexa‐647 anti‐rabbit and Alexa‐488 anti‐mouse fluorescent secondary antibodies. Fluorescent sections were counterstained with DAPI. Experiments with the omission of primary antibodies were used as negative controls.

### 2.3 Assessment strategy for quantitative neuropathological outcomes

Parenchymal microglial/macrophage protein expression (Iba1+, CD68+, TMEM119+), lymphocytes (CD3+ and CD8+), GFAP+ astrocytes, and neuronal density (NeuN+) were quantified in field of views (FOVs) in the grey matter within each cortical layer along predefined and systematically spaced trajectories that were arranged perpendicular to the cortical surface, as previously described [14]. Analyses were restricted to NAGM to avoid the potential confounds of lesional parenchyma. PLP immunostains were used to identify lesional areas using optimized methods [11]. Data on the extent of fibrinogen deposition derived from analyses of adjacently immunolabeled sections to those used in the current study are available [11].

### 2.4 Parenchymal microglia protein expression and GFAP+ astrocyte quantitation

Parenchymal microglial/macrophage protein expression (Iba1+, CD68+, TMEM119+) and GFAP+ astrocyte density were assessed using a semi‐automatic color‐based extraction method and are reported as millions of pixels/mm^2^, using optimized methods [14]. By restricting our analyses to NAGM, we avoided morphological differences in lesional microglia confounding pixel counts. Lymphocytes (CD3+ and CD8+) were manually counted and are reported as cells/mm^2^, as previously described [14].

### 2.5 Neuronal density measures

Motor cortical neuronal density was assessed by manually counting NeuN+ cells that had a nucleus with a single, large, and clearly visible nucleolus (reported as neurons/mm^2^). NeuN+ cells were counted along the same trajectories as in all other analyses.

### 2.6 Quantitation of inhibitory synapses

Inhibitory synapses (GAD+) were quantified in a subset of MS cases (*n* = 20). We selected HLA‐DRB1*15+ (*n* = 10) and HLA‐DRB1*15− (*n* = 10) MS cases; matched for age, sex, post mortem interval, disease duration, and cortical layer 5 neuronal density. We matched the cohorts for disease duration and layer 5 neuron density to minimize confounds associated with the disease stage and the extent of neurodegeneration. Control cases were also assessed (*n* = 7). Clinical details of the cohort can be found in Table 3.

**TABLE 3:** Clinical details of MS and control cohort used in analysis of inhibitory synapses

	MS cases (*n* = 20)	*HLA*‐DRB1*15+ (*n* = 10)	*HLA*‐DRB1*15− (*n* = 10)	Controls (*n* = 7)
Age of death (yr)	60.5 (range: 42–82)	60.7 (range: 42–82)	60.2 (range: 48–80)	78 (range: 68–91)
Duration of disease (yr)	28.1 (range: 11–58)	27.4 (range: 12–58)	28.9 (range: 11–38)	n/a
Sex	M = 2; F = 18	M = 1; F = 9	M = 1; F = 9	M = 4; F = 3
PM interval (h)	18.63 (range: 7–38)	18.8 (range: 7–38)	18.45 (range: 7.5–28)	28.4 (range: 10–52)
Cortical layer 5 neuronal density (NeuN+ cells/mm^2^)	358 (range: 138–595)	339 (range: 229–595)	377 (range: 138–555)	338 (range: 298–368)

Note

Values represent the mean unless stated otherwise.

Abbreviation: n/a, not applicable; PM, post‐mortem.

MS cases and controls double‐labeled with GAD and neurofilament were imaged using an Olympus FV1000 confocal system mounted on an Olympus IX81 microscope using an Olympus 60X 1.4 NA oil immersion lens. The image acquisition parameters were held constant for all images captured regardless of disease or genotype status (see Table S1 for full details of technical parameters). As a result of the fact that time in formalin, post‐mortem interval, tissue pH, agonal state, and cause of death can all influence immunofluorescence, a light intensity normalization method was applied.

Analysis of inhibitory synapses was restricted to pyramidal neurons in cortical layer 5 NAGM. Analysis was restricted to NAGM to avoid the potential confounds of lesional parenchyma. Cortical layer 5 was selected for its relevance to MS progression given the long axonal projections of this layer in the corticospinal system. While the neuronal loss in cortical layer 5 is minimal compared to controls, we previously reported a deleterious role of parenchymal fibrinogen deposition on layer 5 neuronal survival [11]. Fibrinogen deposition was, therefore, factored into analyses, as relevant. Pyramidal neurons that showed clear evidence of inhibitory synapses were sampled by moving the microscope stage systematically through layer 5 of the motor cortex until 30–40 neurons could be imaged for each case in the areas of NAGM.

#### 2.6.1 Creation of novel quantitation method to estimate inhibitory synaptic coverage

**FIGURE 1:**
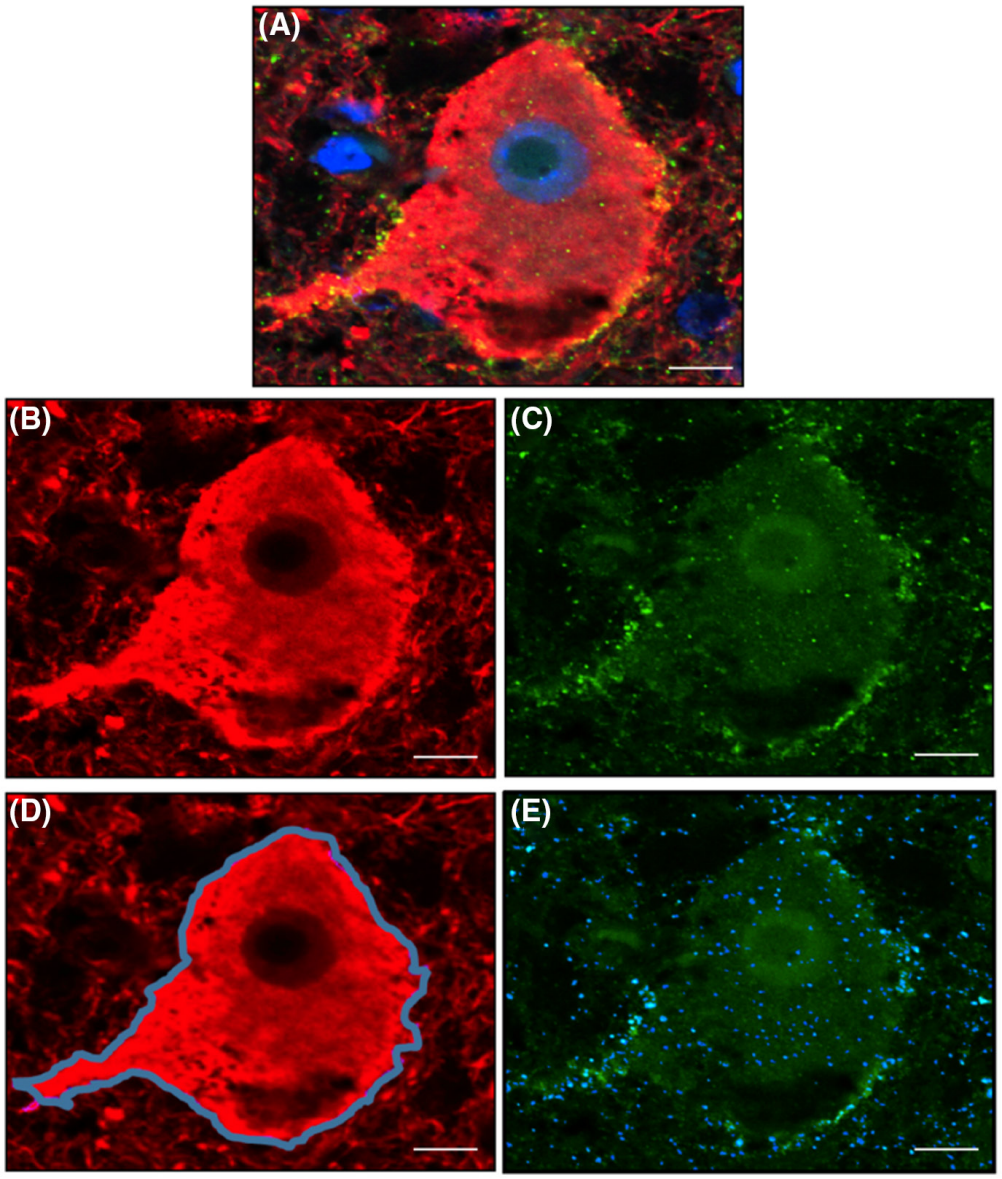
Quantitation of inhibitory synaptic coverage of cortical layer 5 neurons. Confocal images were acquired of cortical layer 5 pyramidal neurons double‐labeled for neurofilament (neurons; red) and GAD 65/67 (GAD+ synapses; green), and counter‐stained with DAPI (nuclei; blue) (A). Acquired images were split into their respective color channels (B and C) before being subjected to a segmentation algorithm optimized in collaboration with colleagues at the Institute of Biomedical Engineering (Oxford). Neuronal perikarya (D; blue outline) and GAD+ synapses (E; blue overlay) were outlined and quantified as outlined in the main text. Scale bar represents 10 μM

We defined inhibitory synaptic coverage (*C*′) as:

(1) $$C^{\prime }=\frac{N_{s}}{A\left(d\right)}$$

where *N_s_* is the number of synapses in a region defined by a segmented neuron, and *A*(*d*) is the area of that segmented neuron. The use of the number of synapses instead of the synaptic area mitigated difficulties in establishing the boundaries of the synapse. Based on our definition of inhibitory synaptic coverage outlined in (1), the algorithm for its computation was divided into three parts: (i) Segmentation of the neuron boundary, (ii) segmentation of synapses, and (iii) combination of the two results to compute the coverage (Figure 1). Using this computational algorithm, inhibitory synaptic density was assessed in each case by quantifying the extent of GAD+ synapses overlapping with the neuronal membrane (expressed as GAD+ synapses/μm^2^ neuronal membrane). Analyzed images were quality controlled and neurons that failed to optimally segment were deleted.

In addition to inhibitory synapses, the size of the neurons that expressed inhibitory synapses was also calculated, by summating the area within the segmented neuronal boundary with the area of the delineated boundary (expressed as μm^2^). Both inhibitory synaptic coverage and neuronal size were validated with manual delineation using ImageJ software.

### 2.7 Assessment of synaptophysin

To investigate global synaptic changes, synaptophysin was assessed in MS cases that had “high” levels of inhibitory synapses (*n* = 3) and “low” levels of inhibitory synapses (*n* = 3), matched for age, sex, PM interval, and disease duration. Analysis of synaptophysin coverage utilized the same workflow as that used for assessment of inhibitory synapses, as discussed above, and are expressed as syn+/μm^2^.

### 2.8 Statistical analyses

Relationships between continuous pathologic variables were assessed using Spearman rank correlation coefficients. To evaluate the predictive value of microglial protein expression on neuronal density, multiple linear regression models were constructed, controlling for age, sex, and post‐mortem interval. As we have previously shown that fibrinogen deposition inversely correlates with neuronal density in the MS cortex [11], we additionally corrected for fibrinogen deposition in these analyses. Linear regression models were fitted to evaluate the influence of MS disease status, as well as HLA‐DRB1*15 status *within* MS cases, on continuous pathological outcome measures while controlling for age, sex, and post‐mortem interval. The distribution of data was assessed analytically and graphically and transformed as required. Data are presented ±SEM. All tests of hypotheses were carried out using two‐sided tests. In all analyses, *p*‐values less than 0.05 were considered significant. Statistical analyses were carried out using SPSS v21 and v26 software.

## 3 RESULTS

### 3.1 Clinical features of MS cases and controls

Clinical details for the MS and control cohort used in this study can be found in Table 1, with details for the subset of MS and control cases included in the analysis of inhibitory synapses found in Table 3. Most MS cases were classified as secondary progressive. The cause of death in our control cohort included metastatic colon cancer, heart failure, myocardial infarction, esophageal carcinoma, bronchopneumonia, vascular ischemia, metastatic gastric cancer, and gastrointestinal bleeding. Unfortunately, two cases in our cohort did not have a clinical cause of death documented. Control cases did not have a diagnosis, or pathological evidence, of neuroinflammatory disease.

### 3.2 Neuropathological findings: MS versus controls

#### 3.2.1 Parenchymal immune cells

Representative staining of different immunohistochemical markers are demonstrated in Figure S1. The number of lymphocytes was greater in MS compared to control (CD3: MS: 4.16 ± 0.62 cells/mm^2^ vs. control: 0.88 ± 0.25 cells/mm^2^, *p* < 0.001; CD8: MS: 1.77 ± 0.31 cells/mm^2^ vs. control: 0.1 ± 0.07 cells/mm^2^, *p* < 0.001). Microglial protein expression was not greater in MS when compared to the current control cohort (Iba1: MS: 6.18 ± 0.55 millions of pixels/mm^2^ vs. control: 6.16 ± 0.7 cells/mm^2^, *p* = 0.979; CD68: MS: 1.77 ± 0.32 millions of pixels/mm^2^ vs. control: 1.35 ± 0.32 cells/mm^2^, *p* = 0.368; TMEM119: MS: 0.47 ± 0.23 millions of pixels/mm^2^ vs. control: 0.49 ± 0.23 cells/mm^2^, *p* = 0.9).

#### 3.2.2 Parenchymal GFAP+ astrocytes

GFAP+ astrocyte coverage was not significantly increased in MS compared to controls (MS: 0.85 ± 0.39 million pixels/mm^2^ vs. control: 0.84 ± 0.43 million pixels/mm^2^, *p* = 0.583).

#### 3.2.3 Neuronal density

In total, ~65,000 neurons were manually counted in the MS cohort, and ~5000 neurons were manually counted in controls. Neuronal density did not differ between MS and control cases (MS: 414 ± 17 neurons/mm^2^ vs. control: 431 ± 27 neurons/mm^2^, *p* = 0.909).

#### 3.2.4 Relationships between parenchymal immune cells and neuronal density

In MS and controls, relationships between microglial protein expression, lymphocytes, GFAP+ astrocyte coverage, and neuronal density were explored.

##### Microglial protein expression and neuronal density

**FIGURE 2:**
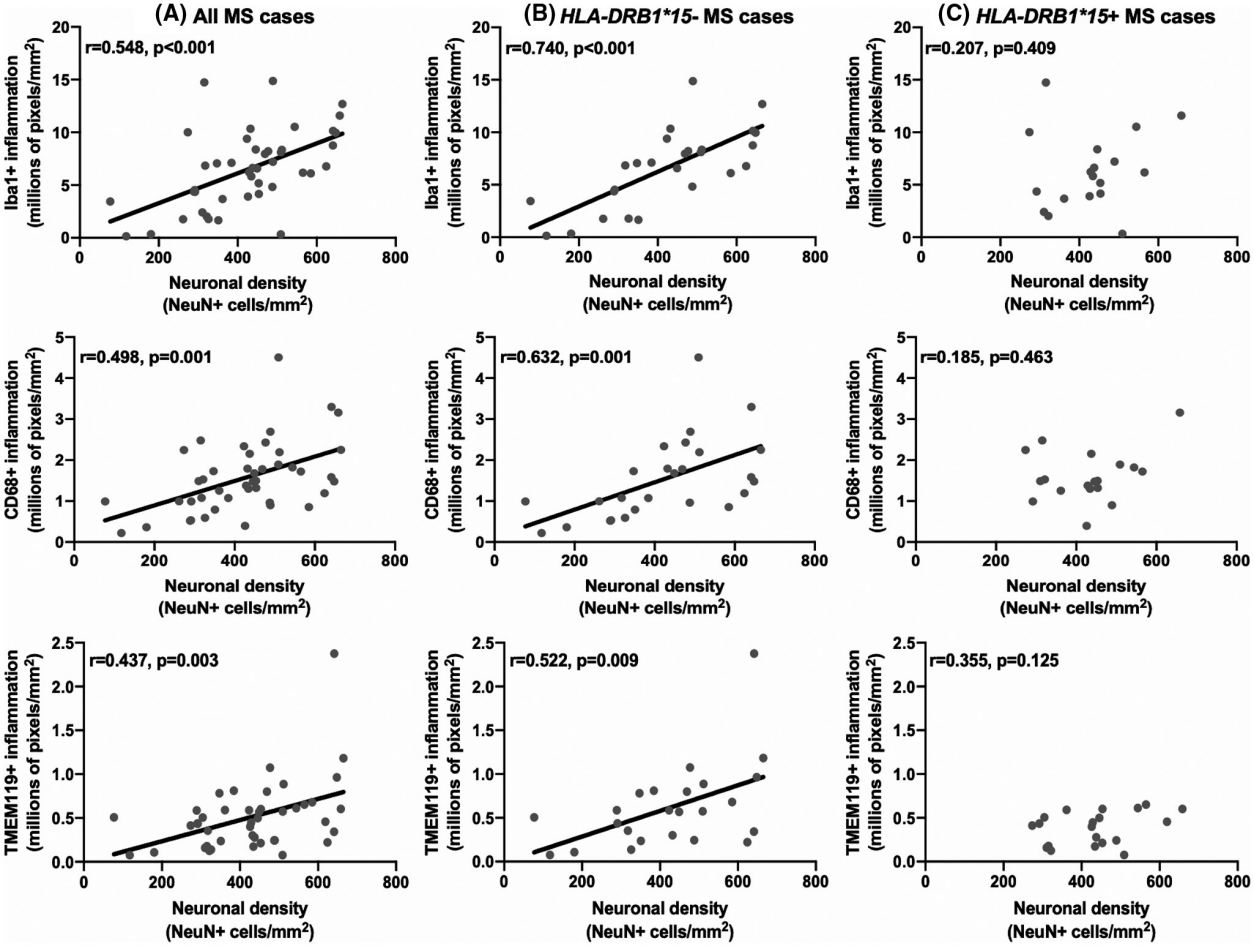
Relationship between microglial expression and neuronal density in the MS cortex. Microglial expression positively correlated with neuronal density when all MS cases were considered (A). This finding was restricted to *HLA*‐DRB1*15− MS cases (B). No relationship between microglial expression and neuronal density was detected in *HLA*‐DRB1*15+ MS cases (C)

In MS, microglial/macrophage protein expression (total, i.e., Iba1+, and activated, i.e., CD68+) positively correlated with neuronal density (Iba1+: *r* = 0.548, *p* < 0.001, CD68+: *r* = 0.498, *p* = 0.001). A positive correlation between TMEM119+ resident microglia and neuronal density was also observed (*r* = 0.437, *p* = 0.003) (Figure 2A). In control cases, no relationship between microglial/macrophage protein expression and neuronal density was seen (Iba1+: *r* = 0.07, *p* = 0.88; CD68+: *r* = 0.33, *p* = 0.47; TMEM119+: *r* = 0.26, *p* = 0.66) (Figure S2).

##### Lymphocytes and neuronal density

In MS, no relationship was observed between lymphocytes and neuronal density (CD3+: *r* = −0.074, *p* = 0.626; CD8+: *r* = 0.193, *p* = 0.199). In control cases, no relationship between lymphocytes and neuronal density was seen (CD3+: *r* = −0.216, *p* = 0.641; CD8+: *r* = −0.134, *p* = 0.775).

##### GFAP+ astrocytes and neuronal density

In MS, no relationship was observed between GFAP+ astrocytes and neuronal density (*r* = 0.196; *p* = 0.192). In control cases, no relationship between GFAP+ astrocytes and neuronal density was seen (*r* = −0.143; *p* = 0.760).

### 3.3 HLA‐DRB1*15 status influences the relationship between microglial protein expression and neuronal density

We have previously shown greater microglial protein expression (Iba1+, CD68+) in the cortex of HLA‐DRB1*15+ MS cases that died at younger ages [14]. In the current study, we extend these findings by demonstrating the extent of TMEM119+ resident microglial expression was reduced in HLA‐DRB1*15+ MS cases compared to HLA‐DRB1*15− MS cases, after correcting for age, sex, post‐mortem interval, and neuronal density (*15*+: 0.3833 ± 0.19 million pixels/mm^2^ vs. *15*−: 0.6184 ± 0.49 million pixels/mm^2^, *p* = 0.031). A strong positive correlation between CD68+ and TMEM119+ microglia was observed, but only in HLA‐DRB1*15− MS cases (*r* = 0.68, *p* = 0.0002) (Figure S3).

HLA‐DRB1*15 status did not influence neuronal density in MS cases (*15*+: 417 ± 20 neurons/mm^2^ vs. *15*−: 411 ± 26 neurons/mm^2^, *p* = 0.766). HLA‐DRB1*15 status impacted the relationship *between* microglial protein expression and neuronal density. Statistically, these relative relationships are not contingent on absolute differences between groups. HLA‐DRB1*15− MS cases demonstrated a positive correlation between microglia/macrophages and neurons (Iba1+: *r* = 0.740, *p* = 0<0.001; CD68+: *r* = 0.632, *p* = 0.001). These findings were confirmed to be specific to resident microglia in HLA‐DRB1*15− MS cases (TMEM119+: *r* = 0.522, *p* = 0.009) (Figure 2B). In contrast, no relationships between microglia/macrophages and neurons were detected in HLA‐DRB1*15+ MS cases (Iba1+: *r* = 0.207, *p* = 0.409; CD68+: *r* = 0.185, *p* = 0.463; TMEM119+: *r* = 0.355, *p* = 0.125) (Figure 2C). HLA‐DRB1*15 genotype status did not impact relationships between lymphocytes (CD3+, CD8+), or GFAP+ astrocyte coverage, and neurons (data not shown).

The striking differences in microglial‐neuron relationships observed between HLA‐DRB1*15 genotype groups prompted further exploration using multiple linear regression analyses. We have previously shown that fibrinogen deposition inversely correlates with neuronal density in the MS cortex [11]. We, therefore, sought to confirm that the genotype‐dependent relationships between microglia and neurons outlined above remained after correcting for fibrinogen deposition, in addition to age, sex, and post‐mortem interval. In HLA‐DRB1*15− MS cases microglia positively correlated with neurons, a finding not observed in HLA‐DRB1*15+ MS cases, after correction for fibrinogen deposition, age, sex, and post‐mortem interval (HLA‐DRB1*15− MS cases: Iba1+: *β* = 0.549, *p* = 0.005; CD68+: *β* = 0.544, *p* = 0.004; TMEM119+: *β* = 0.438, *p* = 0.036; HLA‐DRB1*15+ MS cases: Iba1+: *β* = −0.266, *p* = 0.422; CD68+: *β* = −0.178, *p* = 0.582; TMEM119+: *β* = 0.168, *p* = 0.513).

### 3.4 Inhibitory synapses are selectively reduced in MS motor cortical neurons

A total of 883 neurons were photographed in MS cases and controls. A representative image of neurofilament fluorescence is depicted in Figure S4. After image quality control, 239 (27%) were deleted because of: (i) poor image contrast (*n* = 123), (ii) erroneous capture of multiple processes in the segmentation (*n* = 14), (iii) incorrect neuron captured (*n* = 4), and (iv) two neurons segmented in same image (*n* = 1). Therefore, a total of 644 neurons were analyzed (MS, *n* = 492: HLA‐DRB1*15+, *n* = 252; HLA‐DRB1*15−, *n* = 240; and control, *n* = 152).

**FIGURE 3:**
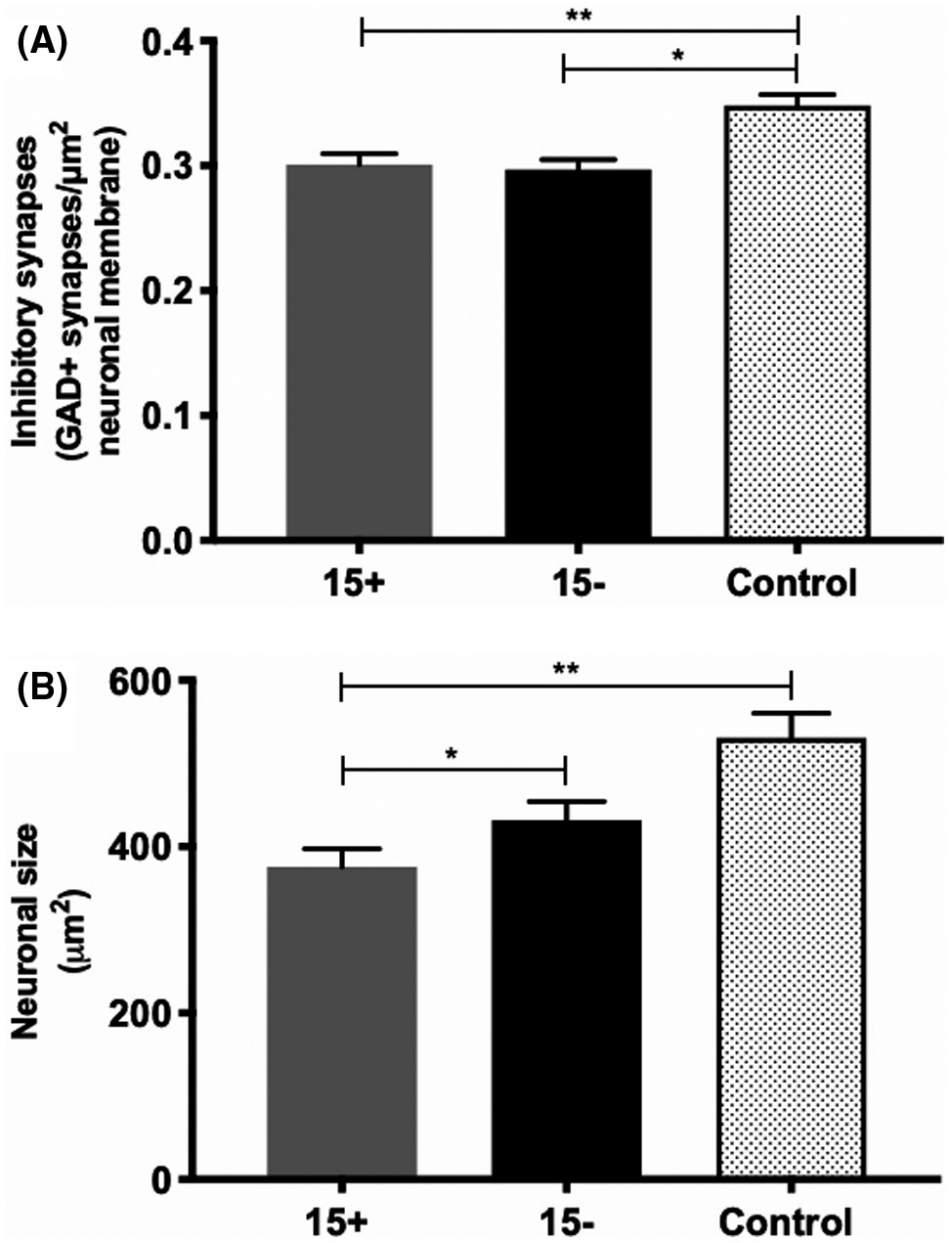
Inhibitory synapses and neuronal size in MS and control. Inhibitory synapses were reduced in MS, regardless of *HLA*‐DRB1*15 genotype status (A). Pyramidal neurons expressing inhibitory synapses in cortical layer 5 were of a smaller area in MS, particularly in the *HLA*‐DRB1*15+ cohort (B). *p < 0.05; **p < 0.01

We detected a 14% reduction in the density of inhibitory synapses in MS compared to control (MS: 0.299 ± 0.006 GAD+ synapses/μm^2^ neuronal membrane vs. control: 0.348 ± 0.009 GAD+ synapses/μm^2^ neuronal membrane, *p* = 0.005) (Figure 3A) with no differences detected between HLA‐DRB1*15 genotype groups (*15*+: 0.3 ± 0.008 GAD+ synapses/μm^2^ neuronal membrane vs. *15*−: 0.297 ± 0.008 GAD+ synapses/μm^2^ neuronal membrane, *p* = 0.493) (Figure 3A). No relationships between inhibitory synaptic density and neuronal density or microglial protein expression were found in control or MS groups, irrespective of HLA‐DRB1*15 status in the latter (data not shown). The observed reduction of inhibitory synapses in MS cases was not confounded by a global reduction in synapse coverage as assessed by synaptophysin (high inhibitory synaptic coverage: 0.514 ± 0.037 syn+/μm^2^ vs. low inhibitory synaptic coverage: 0.484 ± 0.012 syn+/μm^2^, *p* = 0.48).

### 3.5 Neurons expressing inhibitory synapses are smaller in MS, particularly in HLA‐DRB1*15+ cases

Neuronal area was reduced by 24% in MS compared to control (MS: 403 ± 15 μm^2^ vs. control: 531 ± 29 μm^2^, *p* = 0.001). HLA‐DRB1*15 status influenced neuronal size. Neurons from HLA‐DRB1*15+ MS cases were 13% smaller than those in their HLA‐DRB1*15− counterpart (*15*+: 376 ± 21 μm^2^ vs. *15*−: 432 ± 22 μm^2^, *p* = 0.018). When HLA‐DRB1*15+ and HLA‐DRB1*15− MS cases were assessed separately, only HLA‐DRB1*15+ neurons demonstrated a reduction in neuron size relative to control (*15*+: *p* < 0.001; *15*−: *p* = 0.149) (Figure 3B). Neuronal size did not relate to neuronal density, inhibitory synaptic coverage, or microglial protein expression in MS or control groups. In MS cases, HLA‐DRB1*15 status did not impact these relationships (data not shown).

## 4 DISCUSSION

Unraveling the determinants of cortical MS pathology will be essential to preventing irreversible neurological disability that is characteristic of progressive disease. Despite demyelination and chronic inflammation that can last decades, cortical neuronal loss in MS is surprisingly scant. Our findings implicate microglia in a complex relationship with neurodegeneration. We demonstrate that microglia protein expression positively correlates with neuronal density in motor cortical NAGM, but that this relationship is lost in cases carrying the HLA‐DRB1*15 allele. A deleterious impact of HLA‐DRB1*15 on neuronal health is substantiated by evidence of more neuronal atrophy in MS cases harboring this allele. The selective reduction of inhibitory synapses in MS may be a key protective factor. These findings implicate the main genetic risk factor for MS, HLA‐DRB1*15, in relationships relevant to disease progression, and provide evidence of disease‐related changes to inhibitory synapses that are relevant to cortical neuronal survival.

### 4.1 The relationship between microglia and neurons is modulated by HLA‐DRB1*15 status

In our series, we found that motor cortical NAGM neurons were positively associated with microglial protein expression, but only in HLA‐DRB1*15− cases. This finding is consistent with the notion that inflammation is more deleterious in HLA‐DRB1*15+ cases, and further exemplifies the contribution of genotype to immune phenotypes in MS [14, 16, 17]. Altogether, our data add to the wealth of experimental evidence that has shown marked phenotypic heterogeneity in microglial inflammation, with these cells implicated in both neurodegenerative and neuroprotective processes. Various reports have noted microglia‐mediated reactive oxygen [18], protease [19], and inflammatory cytokine secretion [20], recruitment and activation of lymphocytes [21], and cytotoxicity [22]. Other studies have demonstrated microglial‐mediated promotion of axon [23] and myelin regeneration [24], clearance of cellular debris [25], and secretion of neurotrophic factors [26]. Our results show that TMEM119+ resident microglia are positively correlated with neuronal density in HLA‐DRB1*15− MS cases. We also detect an increase in TMEM119+ expression in HLA‐DRB1*15− MS cases compared with HLA‐DRB1*15+ MS cases. TMEM119+ resident microglia have been recently shown to inversely associate with lymphocytic and lymphocyte‐derived cytokines in the brain parenchyma in MS [27]. A preponderance of TMEM119+ resident microglia in HLA‐DRB1*15− MS cases may, therefore, explain why positive associations between microglia and neurons are observed only within this genotype group. In contrast, carriage of HLA‐DRB1*15 may lead to greater extravasation of peripheral macrophages into the CNS that, through ill‐defined mechanisms, lead to diffuse tissue damage [28].

### 4.2 Selective loss of inhibitory synapses in the MS cortex

We found that inhibitory synapses are selectively lost in the MS motor cortical NAGM. This finding is consistent with previous reports that have detected disturbed inhibitory neurotransmission in the MS cortex based on transcript analysis of GAD mRNA [29]. In rodents, it has been demonstrated that microglia‐mediated stripping of GAD+ synapses protects neurons from inflammatory damage [13]. GAD catalyzes the decarboxylation of glutamate to GABA, and as such is the primary source of inhibitory neurotransmitters in the CNS. Reduced inhibitory innervation of motor neurons increases firing of synaptic NMDA and reduces cell death by stimulating phosphorylation of CREB and expression of BDNF to prevent apoptosis [13]. While we were unable to relate inhibitory synapses to microglial protein expression or neuronal density, the fact that inhibitory synaptic stripping occurs on time scales measured in days likely renders such relationships difficult to discern in human autopsy material of cases with end‐stage disease [13]. Whether loss of inhibitory synapses is neuroprotective in MS is not clear. Increased neuronal firing will likely increase neuronal stress when ATP synthesis is already reduced through oxidative injury and mitochondrial dysfunction, as has been demonstrated in the MS cortex [30]. Recent work has added further to this complexity by demonstrating a role for complement in the selective loss of inhibitory synapses from demyelinated hippocampus that positively associates with cognitive impairment [31]. While the reduction of inhibitory synapses in our cohort occurred in the apparent absence of substantial neuronal loss, we hypothesize that functionally relevant changes to cortical neurons likely extend beyond overt loss of cellularity.

### 4.3 Neuronal atrophy is influenced by HLA‐DRB1*15 status

In the current study, we have demonstrated that neurons in NAGM cortical layer 5 expressing inhibitory synapses are smaller in MS, particularly within the HLA‐DRB1*15+ cohort. This is consistent with the findings of previous studies demonstrating reduced neuronal size in the MS cortex [5], but our work extends this to show an influence of genotype. It has been suggested that a decrease in neuronal size in NAGM is because of axonal degeneration [5], but whether changes to neuronal size correlate with the motor deficit is unknown, and will require additional investigation. However, the apparent deleterious effect of carrying the HLA‐DRB1*15 allele further highlights that parenchymal inflammation has an impact on neuronal morphology, and may play a key role in determining the fate of cortical neurons.

### 4.4 Limitations

Autopsy material is necessarily biased toward the inclusion of MS cases with more severe disability and longer duration of disease than might be ideal in understanding the determinants of neuronal loss. Further, we recognize that pathology can only provide a static view of a dynamic process. However, our systematic objective neuropathological outcome measures go beyond the resolution of current *in vivo* technologies and provide critical insight into disease‐specific processes that animal models cannot capture. We acknowledge that the lack of available high‐quality non‐neurological control tissue may have hindered the detection of more substantial differences in neuronal density coverage compared to MS cases, as previously reported. However, our study evaluated a large number of MS cases, which enabled the detection of important and novel genotype comparisons. Further, the development of a validated computational algorithm to address confounds known to impact immunofluorescence experiments, which are typically overlooked in confocal imaging analyses, provides confidence in the robustness of our positive findings that will likely have a substantial impact on future MS research. Finally, we acknowledge that increased neuronal densities could relate to more pronounced degeneration of neuropil, and vice versa. That said, our interpretations should be viewed in the context of existing clinical, radiographic, and pathologic data that demonstrate HLA‐DRB1*15+ cases have an earlier age of onset and worse outcomes in MS [32, 33, 34]. While this is yet to be conclusively shown by large‐scale genome‐wide association studies (other than the age of onset), existing measures of clinical disease severity likely lack the resolution that post‐mortem quantitative neuropathological outcome measures provide to evaluate the impact of genetic variation on disease outcome. Our data build on previous work from our group and others and provides important insight into the potential mechanisms by which HLA‐DRB1*15 is able to influence the MS disease process.

## 5 CONCLUSIONS

We show that the chronic microglial inflammatory milieu in the MS cortex is influenced by HLA‐DRB1*15 status. The selective reduction of motor cortical neuronal inhibitory synaptic coverage in MS may also be a key factor in neuronal survival. Taken together, this work highlights that innate inflammation, inhibitory synapses, and HLA‐DRB1*15 may interact to modulate neuronal physiology in the MS cortex. This work also highlights that carrying HLA‐DRB1*15 may associate with microglial dysfunction, providing insight into the cellular mechanisms underpinning MS severity. These findings challenge the prevailing view that microglia, as the resident immune cell population of the brain, act as perpetrators of pathology rather than protectors. In so doing, our observations have relevance not only to MS, but also to other neurodegenerative diseases.

In summary, our work emphasizes that the examination of human tissue can still reveal surprising associations that challenge our views on pathogenesis, and that underlying genetic heterogeneity has an important influence on pathology that remains overlooked in our treatments. Future studies aimed at addressing the mechanisms by which genetic variation at the *HLA*‐*DRB1* locus influences the interplay between cortical inflammation and neurodegeneration are urgently required. If successful, therapeutic targets may be identified to promote neuronal survival and by extension, halt the accumulation of irreversible disability in progressive MS.

## CONFLICT OF INTEREST

Nothing to report.

## AUTHOR CONTRIBUTIONS

Richard L. Yates, Jacqueline Palace, Margaret M. Esiri, and Gabriele C. DeLuca conceived and designed the study. Richard L. Yates, Jonathan Pansieri, and Sydney A. Yee acquired the data. Richard L. Yates, Jonathan Pansieri, Jack S. Bell, Sydney A. Yee, Qizhu Li, Margaret M. Esiri, and Gabriele C. DeLuca contributed to the drafting of the manuscript. All authors approved the final version and submission of the manuscript

## ETHICAL STATEMENT

All the work presented in the current manuscript has been given relevant ethical approval. Details are available on request.

## Supporting information


**TABLE S1** Confocal microscope settings for fluorescence analysis
**FIGURE S1** Representative immunohistochemistry. Control cases left column. MS cases right column. PLP (A, B), NeuN (C, D), CD68 (E, F), TMEM119 (G, H), Iba1 (I, J), GFAP (K, L). Scale bar 50 μm
**FIGURE S2** No relationship between microglial inflammation and neuronal density in the control cortex. Microglial inflammation is not correlated with neuronal density in controls using (A) TMEM119 (B) Iba1 and (C) CD68 markers. Note that due to tissue availability, *n* = 6 controls were used for TMEM119+ analyses. *N* = 7 control cases were available for NeuN analyses due to suboptimal NeuN antigenicity in 3 control cases
**FIGURE S3** Relationship between microglial inflammation markers in MS cortex. TMEM119+ microglial inflammation positively correlated with CD68+ microglial inflammation and is restricted to HLA‐DRB1*15‐ MS cases (A). No relationship was detected in HLA‐DRB1*15+ MS cases (B)
**FIGURE S4** Representative neurofilament and GAD immunofluorescence. Confocal images of layer 5 pyramidal neurons counter stained with DAPI (nuclei ; blue, A) are labelled for GAD (GAD ; green, B) and neurofilament (NF ; red, C) at 200x magnification, and representative merge image (D). GAD+ presynaptic contacts are illustrated in the insert (green dots shown by arrows, D) on the surface of NF+ neurons (red). Scale bar 100 µm

## Data Availability

The data that support the findings of this study are available from the corresponding author upon reasonable request.
