# Supplementary material for: The influence of HLA‐DRB1*15 on the relationship between microglia and neurons in multiple sclerosis normal appearing cortical grey matter
Source: Brain Pathol. 2021 Dec 13;32(4):e13041. doi: 10.1111/bpa.13041 (PMC9245937; doi:10.1111/bpa.13041)
Supplement: Supplementary file 1 — TABLE S1 Confocal microscope settings for fluorescence analysis FIGURE S1 Representative immunohistochemistry. Control cases left column. MS cases right column. PLP (A, B), NeuN (C, D), CD68 (E, F), TMEM119 (G, H), Iba1 (I, J), GFAP (K, L). Scale bar 50 μm FIGURE S2 No relationship between microglial inflammation and neuronal density in the control cortex. Microglial inflammation is not correlated with neuronal density in controls using (A) TMEM119 (B) Iba1 and (C) CD68 markers. Note that due to tissue availability, n = 6 controls were used for TMEM119+ analyses. N = 7 control cases were available for NeuN analyses due to suboptimal NeuN antigenicity in 3 control cases FIGURE S3 Relationship between microglial inflammation markers in MS cortex. TMEM119+ microglial inflammation positively correlated with CD68+ microglial inflammation and is restricted to HLA‐DRB1*15‐ MS cases (A). No relationship was detected in HLA‐DRB1*15+ MS cases (B) FIGURE S4 Representative neurofilament and GAD immunofluorescence. Confocal images of layer 5 pyramidal neurons counter stained with DAPI (nuclei ; blue, A) are labelled for GAD (GAD ; green, B) and neurofilament (NF ; red, C) at 200x magnification, and representative merge image (D). GAD+ presynaptic contacts are illustrated in the insert (green dots shown by arrows, D) on the surface of NF+ neurons (red). Scale bar 100 µm [file BPA-32-e13041-s001.docx]

**The influence of *HLA-DRB1*15* on the relationship between microglia and neurons in multiple sclerosis normal appearing cortical grey matter: Supplementary Materials**

Richard L. Yates, BM BCh, DPhil,^1^ Jonathan Pansieri, PhD,^1^ Qizhu Li, MEng,^2^ Jack S. Bell, BM BCh,^3^ Sydney A. Yee, BS,^1^ Jacqueline Palace, DM, BM, FRCP,^1^ Margaret M. Esiri, DM, FRCPath,^1^ Gabriele C. DeLuca, MD, DPhil, FRCPath^1^

^1^Nuffield Department of Clinical Neurosciences, University of Oxford, Oxford, UK, OX3 9DU

^2^Department of Engineering Science, University of Oxford, Parks Road, Oxford, UK, OX1 3PJ

^3^Salford Royal NHS Foundation Trust, Salford, UK

**Running title:** Microglia and neurons in multiple sclerosis

Corresponding Author:

Gabriele C. DeLuca

Nuffield Department of Clinical Neurosciences

Level 1, West Wing, John Radcliffe Hospital

Oxford, UK, OX3 9DU

**Tel:** +44 (0)1865 223 047, **Fax:** +44 (0)1865 231 157

**Email:** [gabriele.deluca@ndcn.ox.ac.uk](mailto:gabriele.deluca@ndcn.ox.ac.uk)

**Supplementary Table 1: Confocal microscope settings for fluorescence analysis**

| General parameters | |
| --- | --- |
| Path | None |
| Name | LiveStop |
| Scan mode | XY |
| System Name | Fluoview FV1000 |
| System Version | 4.2.1.20 |
| X dimension | 1024, 0.0 - 211.761 [um], 0.207 [um/Pixel] |
| Y Dimension | 1024, 0.0 - 211.761 [um], 0.207 [um/Pixel] |
| Channel Dimension | 2, 1.0 - 2.0 [Ch] |
| Image size | 1024 * 1024 [Pixel] |
| Image Size(Unit Converted) | 211.761 [um] * 211.761 [um] |
| Bits/pixel | 12 [bits] |
| Acquisition | |
| Device | FV1000 |
| Observation Mode | LSM |
| Objective Lens | PLAPON60XOSC2 NA:1.40 |
| Objective Lens Magnification | 60X |
| Numerical Aperture | 1.4 |
| Scan Mode | XY |
| Scan direction | One way |
| Sampling speed | 2.0 [us/Pixel] |
| Confocal | ON |
| C.A. | 110 [um] |
| Sequential mode | line |
| Integration type | Line Kalman |
| Integration count | 2 |
| Region mode | None |
| Find mode | X1 |
| Rotation | 0 deg |
| Pan X | 0 [um] |
| Pan Y | 0 [um] |
| Zoom | x1.0 |
| Laser 1 Wavelength | 405 [nm] |
| Laser 1 Transmissivity | 15.5 [%] |
| Laser 2 Wavelength | 405 [nm] |
| Laser 2 Transmissivity | 10.0 [%] |
| Laser 3 Wavelength | 635 [nm] |
| Line 3 transmissivity |  |
| Channel 1 | |
| Channel name | CHS1 |
| Dye Name | Alexa Fluor 405 |
| Spectrum Seperation on | On |
| PMT Detection Mode | Analog |
| PMT Voltage | 880 V |
| Excitation DM Name | DM405/488/559/635 |
| Excitation DM Position | 4 |
| Excitation Wavelength | 405 nm |
| Emission DM Name | SDM490 |
| Emission DM Position | 5 |
| Emission Wavelength | 422 nm |
| BF Position | 425 nm |
| BF Range | 50 nm |
| Channel 2 | |
| Channel Name | CH3 |
| Dye Name | Alexa Fluor 647 |
| Spectrum Seperation | Off |
| PMT Detection Mode | Analog |
| PMT Voltage | 724 V |
| Excitation DM Name | DM405/488/559/635 |
| Excitation DM Position | 4 |
| Excitation Wavelength | 635 nm |
| Emission DM name | Mirror |
| Emission DM Position | 1 |
| Emission Wavelength | 668 nm |
| BF Name | BA655-755 |

Supplementary Figures:


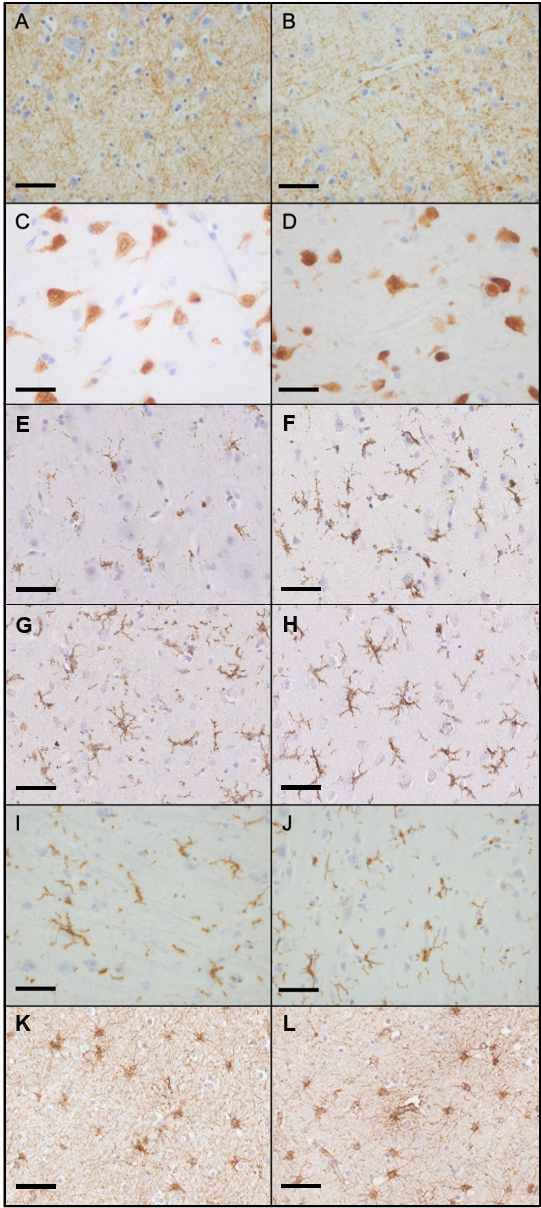


**Figure S1. Representative immunohistochemistry.** Control cases left column. MS cases right column. PLP (A, B), NeuN (C, D), CD68 (E, F), TMEM119 (G, H), Iba1 (I, J), GFAP (K, L). Scale bar 50μm.

*
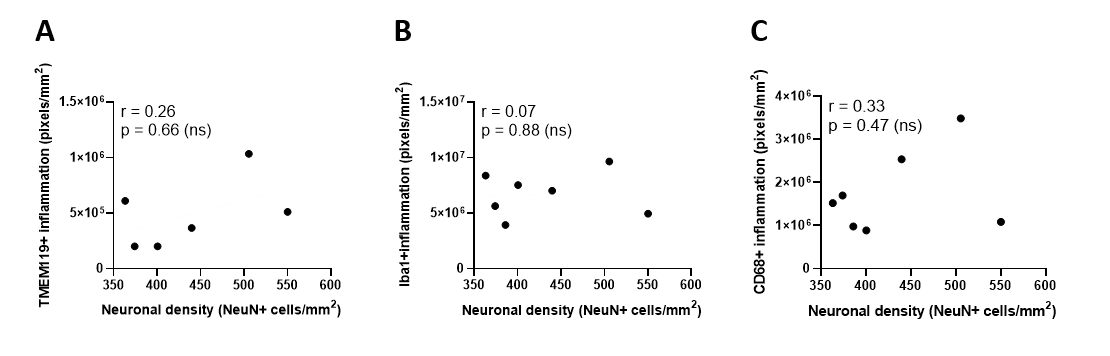
*

**Figure S2. No relationship between microglial inflammation and neuronal density in the control cortex.** Microglial inflammation is not correlated with neuronal density in controls using (A) TMEM119 (B) Iba1 and (C) CD68 markers. Note that due to tissue availability, n=6 controls were used for TMEM119+ analyses. N=7 control cases were available for NeuN analyses due to suboptimal NeuN antigenicity in 3 control cases.


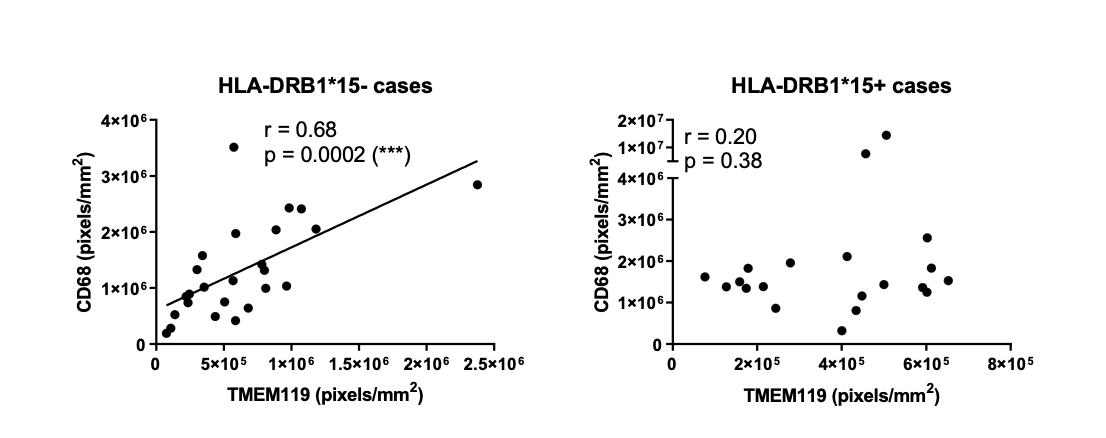


**Figure S3. Relationship between microglial inflammation markers in MS cortex.** TMEM119+ microglial inflammation positively correlated with CD68+ microglial inflammation and is restricted to *HLA-DRB1*15*- MS cases (A). No relationship was detected in *HLA-DRB1*15*+ MS cases (B).

**
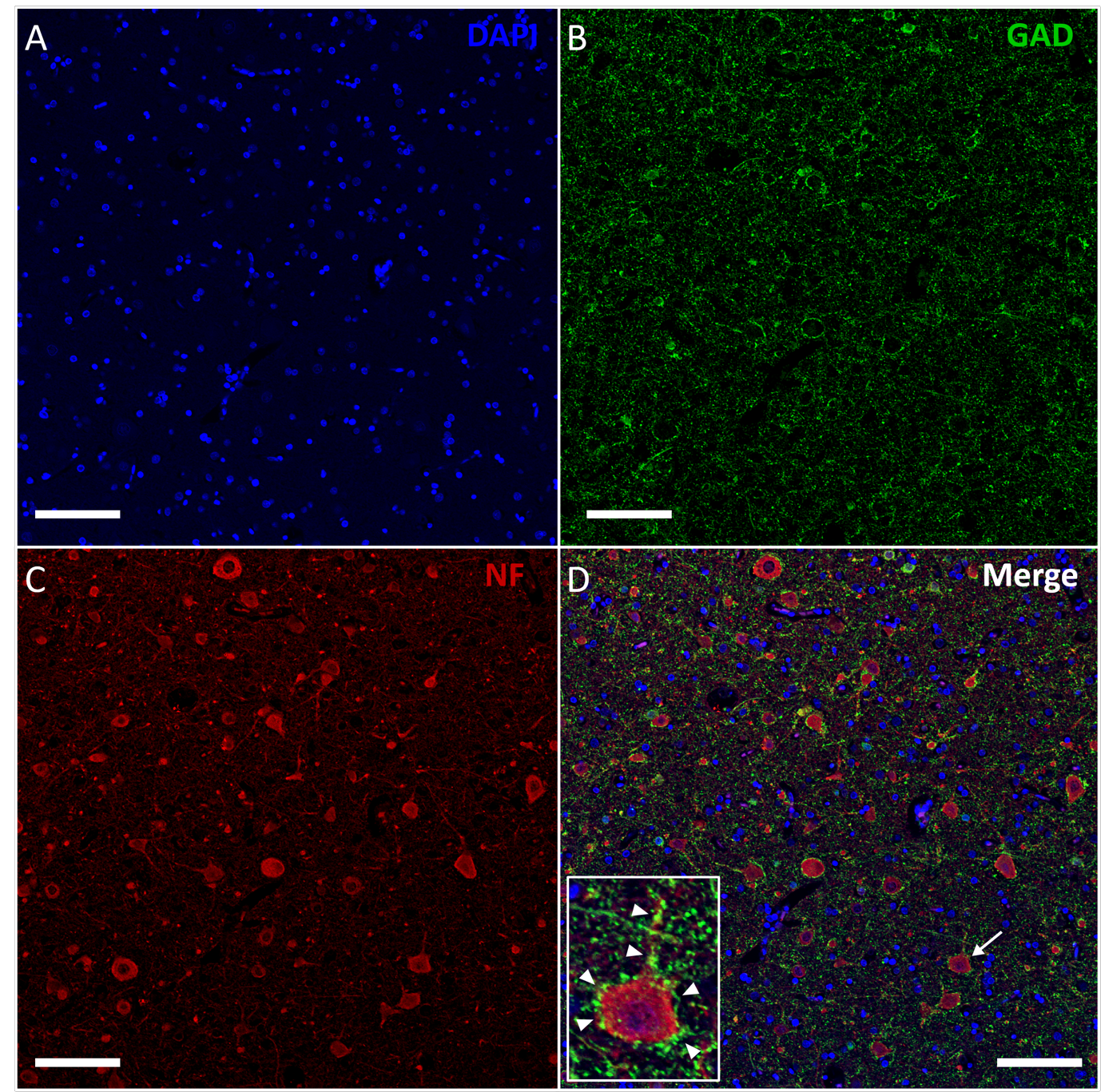
**

**Figure S4. Representative neurofilament and GAD immunofluorescence.** Confocal images of layer 5 pyramidal neurons counter stained with DAPI (nuclei ; blue, A) are labelled for GAD (GAD ; green, B) and neurofilament (NF ; red, C) at 200x magnification, and representative merge image (D).  GAD+ presynaptic contacts are illustrated in the insert (green dots shown by arrows, D) on the surface of NF+ neurons (red). Scale bar 100 µm.
